# Current status of *Helicobacter pylori* resistance to Clarithromycin and Levofloxacin in Malaysia—findings from a molecular based study

**DOI:** 10.7717/peerj.11518

**Published:** 2021-06-09

**Authors:** Suat Moi Puah, Khean Lee Goh, Heng Kang Ng, Kek Heng Chua

**Affiliations:** 1 Department of Biomedical Science, Faculty of Medicine, University of Malaya, Kuala Lumpur, Malaysia; 2 Department of Medicine, Faculty of Medicine, University of Malaya, Kuala Lumpur, Malaysia

**Keywords:** 23S rRNA, *gyrA*, *gyrB*, *H. pylori*, Malaysia, Primary resistance

## Abstract

**Background:**

Resistance to clarithromycin and levofloxacin in *Helicobacter pylori* which resulted in treatment failures has become a major challenge for physicians worldwide. The resistance is mainly mediated by mutations in a specific domain of the 23S rRNA, *gyrA* and *gyrB* genes for clarithromycin and levofloxacin respectively. Hence in this study, we aimed to investigate the current status of *H. pylori* resistance in our hospital to these two antibiotics based on the molecular approach.

**Materials and Methods:**

Gastric biopsy samples were obtained from treatment-naïve patients. Bacterial genomic DNA was extracted using a commercial kit and continued with DNA amplification using polymerase chain reaction (PCR) with specific primers. The PCR amplicons were subjected to sequencing on 23S rRNA gene targeting nucleotide positions at 2,146, 2,147, 2,186 and amino acids at *gyrA* positions 87 and 91 and *gyrB* positions 436, 438, 481, 484 to investigate the possible mutations or polymorphisms of genes that lead to clarithromycin and levofloxacin resistance respectively.

**Results:**

Sixty-one urease-positive gastric biopsy samples were studied. The findings revealed the primary resistance rates to clarithromycin was 14.8% and to levofloxacin was 3.3% in our current scenario based on detection of reported resistance-related mutations of A2147G and D91N in 23S rRNA and *gyrA* genes, respectively. Interestingly, we found a high rate of silent mutations of the *gyrA* codon 87Asn (32.8%, 20/61) and two polymorphisms of the *gyrB* D481E (16.4%, 10/61) and R484K (21.3%, 13/61). The role of these polymorphisms in *gyrB* remained to be elucidated whether the levels of levofloxacin resistance are related to the position/amino acid.

**Conclusion:**

The primary resistance rate of *H. pylori* to clarithromycin has increased compared to the previous report in Malaysia. Therefore, molecular screening could aid and is important for the selection of antibiotics for *H. pylori* eradication therapies.

## Introduction

*Helicobacter pylori* susceptibility to antibiotics is crucial to the treatment success of *H. pylori* eradication treatment regimens. Rising *H. pylori* resistance to clarithromycin has been implicated as the cause of the declining success of the 1-week standard triple therapy which incorporates clarithromycin in the regimen (Buzas & Jozan, 2006; Graham & Fischbach, 2010). Graham and co-workers have persuasively opined that susceptibility testing should be carried out in every case of *H. pylori* infection (Graham & Shiotani, 2008; Graham & Dore, 2016).

Although culturing of the bacterium for susceptibility testing is widely carried out, the success of culture is low even in specialized laboratories (Mégraud et al., 2015). Molecular methods of detecting resistance to antibiotics, on the other hand, is a more robust technique that does not require stringent transport and laboratory requirements for the primary culture of the bacterium. These techniques have now been established with recognized mutations and provide a practical and viable alternative to the traditional methods of testing susceptibility using the culture of the bacterial strains.

Antimicrobial resistance in *H. pylori* is primarily due to the acquisition of point mutations. Thus far, point mutations in 23S rRNA, particularly two specific adjacent nucleotide positions, 2,142 (A to G/C) and 2,143 (A to G), are responsible for most clarithromycin resistance cases observed (Raymond et al., 2007). Using ribosome isolation and erythromycin assays as well as transformation experiments, the role of these two mutations is confirmed involved in the CLA resistance (Binh et al., 2014; Occhialini et al., 1997; Wang & Taylor, 1998; Wang et al., 2020).

For levofloxacin, published data showed that resistance is due to point mutations in the quinolones resistance-determining region (QRDR) of the *gyrA* mainly at codons N87 or D91 (Garcia et al., 2012; Nishizawa & Suzuki, 2014). Using transformation assays, Kim et al. (2005) and Rimbara et al. (2012) demonstrated that resistant transformed *H. pylori* cells harboured the mutation at positions 87 and 91 showed high-level resistance to fluoroquinolone. Besides, the study from Rimbara and co-workers also reported one mutation located closed to QRDR of the *gyrB* at codon E463 conferred norfloxacin resistance in *H. pylori*. In 2006, Miyachi et al. have reported E463 mutation in *gyrB* were detected in highly levofloxacin resistant strains, but occurred together with *gyrA* mutations.

Background testing of *H. pylori* resistance to antibiotics has consistently shown a low prevalence of resistance to clarithromycin and levofloxacin in Malaysia. In the most recent studies, Teh et al. (2014) showed a primary resistance rate 6.8% to both clarithromycin of and levofloxacin. Hanafiah et al. (2019) showed a primary resistance rate of 1.2% and 17.1% to clarithromycin and fluoroquinolones.

In this report, we present our current prevalence of antibiotic resistance to clarithromycin and levofloxacin using molecular-based methods in our local population in Malaysia.

## Materials and Methods

### Gastric biopsy samples collection

The study was performed according to the International Conference on Harmonisation-Good Clinical Practice (ICHC-GCP) guidelines from 1^st^ June 2019–31^st^ May 2020. The study was approved by the ethics committee (MREC ID No: 2019101-7882) of the University of Malaya Medical Centre. The written informed consents were obtained from all participants before gastric biopsy samples were collected from the antrum and body of treatment-naïve patients who were tested *H. pylori*-positive based on the rapid urease test at the endoscopy unit of the University of Malaya Medical Centre. The biopsy samples from the antrum and body of the stomach for each patient were put together and stored in Stuart’s transport medium and kept in a refrigerator at 4 °C until they were sent to the molecular laboratory for analysis.

### Bacterial DNA extraction

Gastric biopsy samples (mixture of antrum and body) obtained were cut into small pieces and bacterial genomic DNA was extracted using QIAmp DNA mini kit (Qiagen, Germantown, MD, USA) according to the manufacturer’s protocol. The extracted DNA was quantitated by using a nano spectrophotometer (Implen, Germany) and stored at −20 °C.

### Molecular detection of known mutations/polymorphisms in *23S rRNA, gyrA* and *gyrB*

According to the reference sequence 26,695 (GenBank accession no. AE000511.1), the primer pairs were designed to detect point mutations/polymorphisms. To detect 23S rRNA gene mutations at positions of 2,146, 2,147 (formerly known as 2,142 and 2,143) and polymorphism at position 2,186 (formerly known as 2,182), forward primer: 5′-ATGAAGCGTTGAATTGAAGC-3′ and reverse primer: 5′-CAACTCTCTTACACTCAGTAAGGC-3′ were employed. To detect mutations associate with levofloxacin resistance, amino acid substitutions at positions 87 (N to L/I/A/K) and 91(D to G/N/A/Y/H) in *gyrA* were carried out using forward primer: 5′CCGAGTTCCACCCATTAG-3′ and reverse primer: 5′ATTTCACTCATCGCGTCTC-3′. For *gyrB*, forward primer: 5′CAATTCGTTAAAGACAGCG-3′ and reverse primer: 5′TTGTAAAGAGGGGCTTGAG-3′ were used to detect mutation at position 463 (E to K/G) and polymorphisms 438 (F to S), 481(D to E/N) and 484 (R to K). DNA amplification was performed using polymerase chain reaction (PCR) with a mixture that contained 60 µL of 10 µM of forward primer, 10 µM of reverse primer, DNA template and RNase-free water using Dream*Taq* polymerase (Thermo Scientific, Waltham, MA, USA). The PCR mixture was denatured at 95 °C for 3 mins, followed by 35 cycles of 95 °C denaturation for 30 s, annealing duration for 30 s (annealing temperature: 23S rRNA—60 °C, *gyrA—*60 °C, *gyrB—*58 °C), and 72 °C extension for 1 min and a final extension cycle, 72 °C for 7 min. The amplicons were electrophoresed on a 1% (w/v) agarose gel, purified and sent for sequencing (First Base Laboratories Sdn. Bhd., Malaysia).

## Results

Sixty-one treatment-naïve gastric biopsy samples were studied. Point mutations/polymorphisms detected which are responsible for primary resistance in *H. pylori* in the present study were listed in Tables 1 and 2. For mutations in the 23S rRNA gene corresponds to the situation of clarithromycin resistance, no A2146G (aka A2142G) mutation was found in this study, but resistance-related mutation A2147G (aka A2143G) and polymorphism T2186C (aka 2,182) were detected with the rate of 14.8% (9/61) and 72.1% (44/61), respectively. Seven samples (11.5%) carried both A2147G and T2186C. For levofloxacin, resistance-related mutation D91N in *gyrA* was detected at a rate of 3.3% (2/61) while E463(G/K) mutation was not found in our study. The polymorphism rates of D481E (16.4%, 10/61) and R484K (21.3%, 13/61) were found in *gyrB*. Silent mutations were detected at base triplet 87 of *gyrA* gene, AAT to AAC (32.8%, 20/61) which do not change the amino acid asparagine. Another similar phenomenon was observed in *gyrB* gene at base triplet 438, whereby seven samples (11.4%) carried a TTC triplet that code for phenylalanine that was similar to wild type. The presence of resistance-related mutations (A2147G and D91N) to the two antibiotics under study was detected in one sample.

**Table 1:** Detection of known mutations present in 23S rRNA, *gyrA* and *gyrB* genes in treatment-naïve patients infected with *Helicobacter pylori*.

Drug	Gene	Mutation point	Forward sequence	Reverse sequence	Frequency (%)
Clarithromycin	23S rRNA	A2147G (aka A2143G)	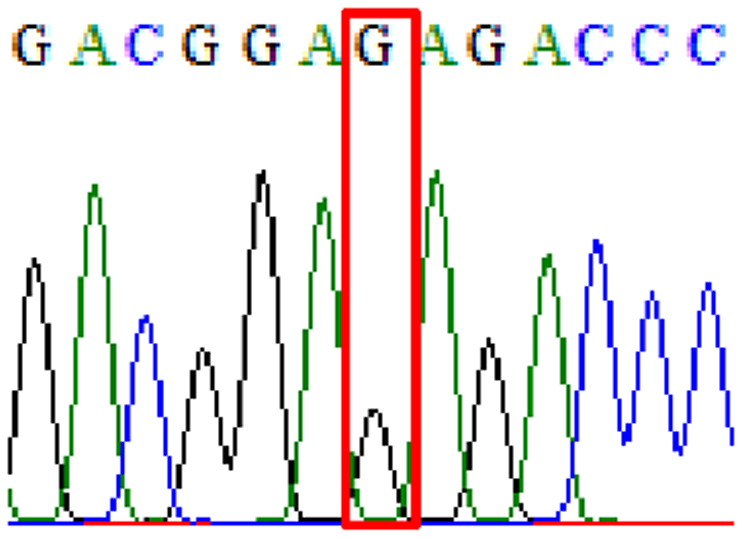	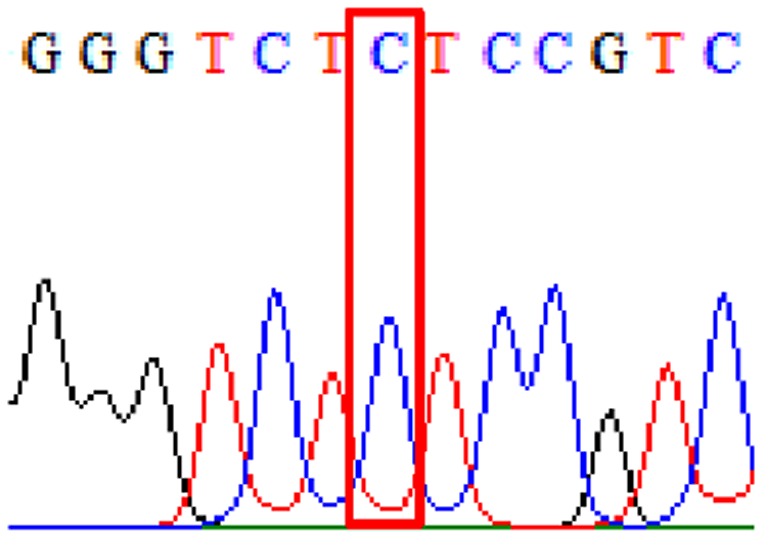	14.8% (9/61)
Levofloxacin	*gyrA*	D91N	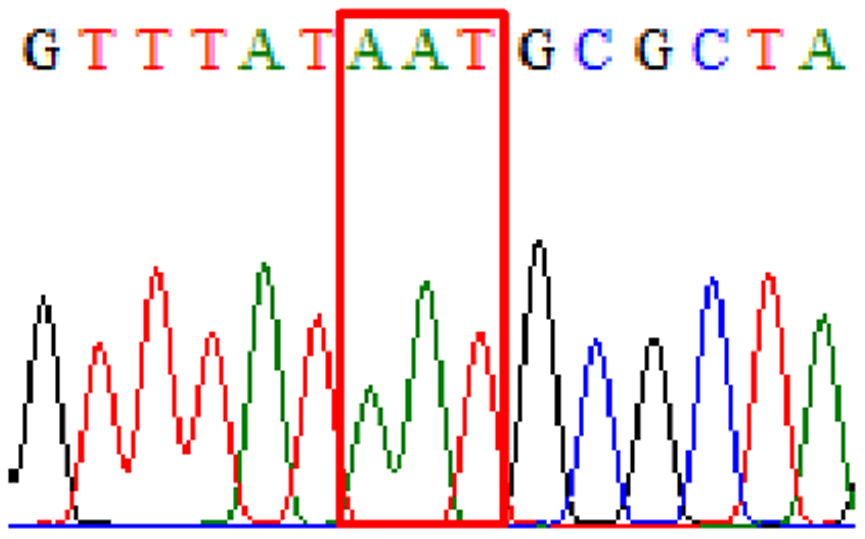	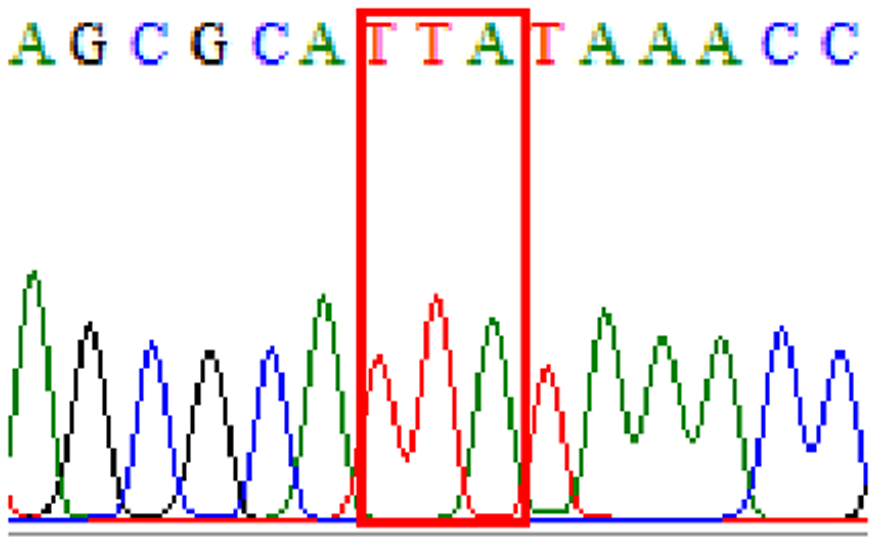	3.3% (2/61)

**Table 2:** Detection of polymorphisms in antibiotic resistance genes (23S rRNA, *gyrA* and *gyrB)* from treatment-naïve patients infected with *Helicobacter pylori*.

Drug	Gene	Polymorphisms	Forward sequence	Reverse sequence	Frequency (%)
Clarithromycin	23S rRNA	T2186C (aka T2182C)	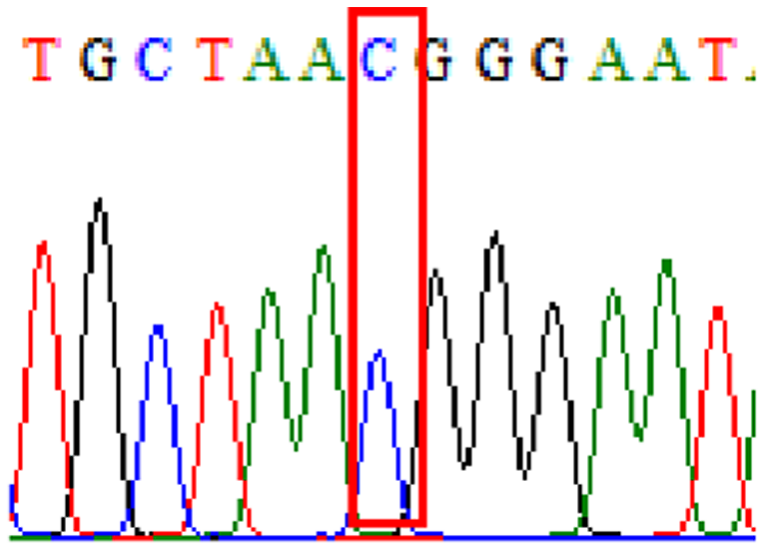	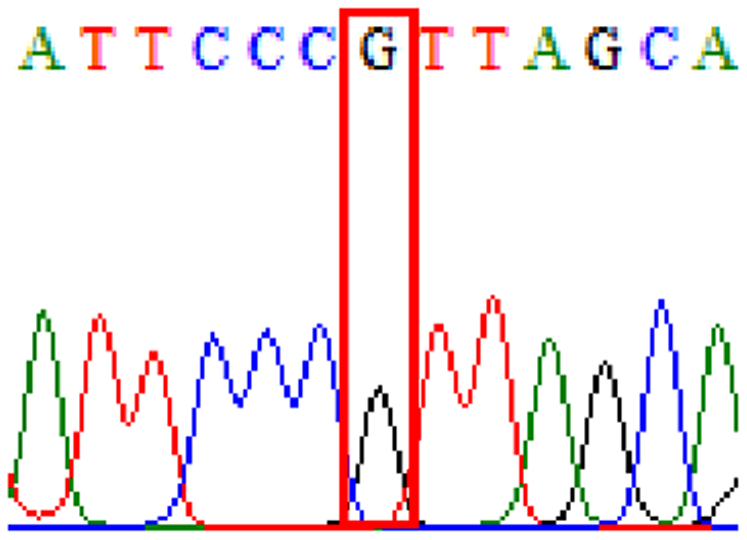	72.1% (44/61)
Levofloxacin	*gyrA*	Silent mutation at 87Asn (AAT to AAC at position 261)	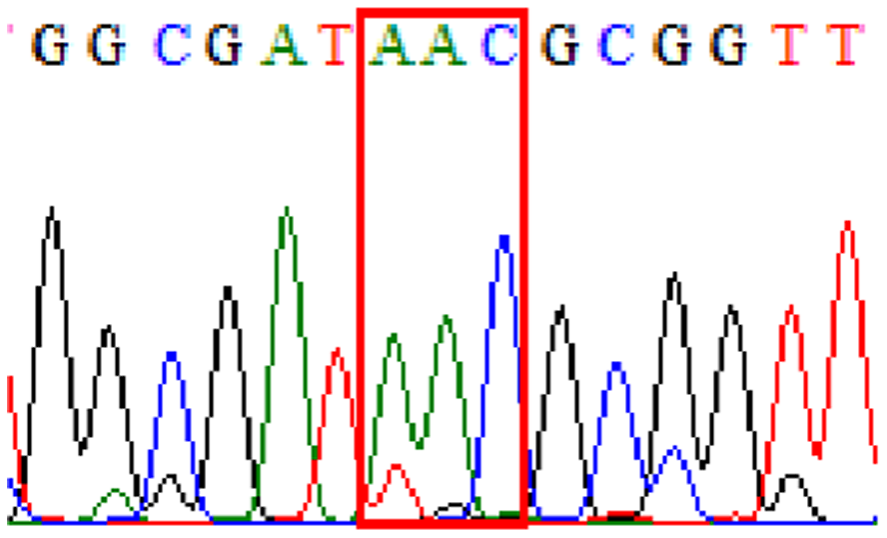	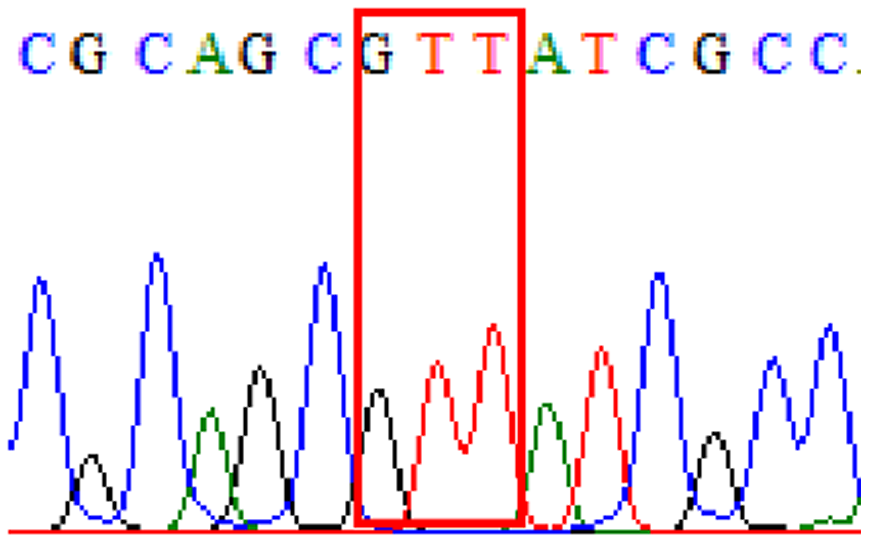	32.8% (20/61)
	*gyrB*	Silent mutation at 438Phe (TTC to TTT at position 1314)	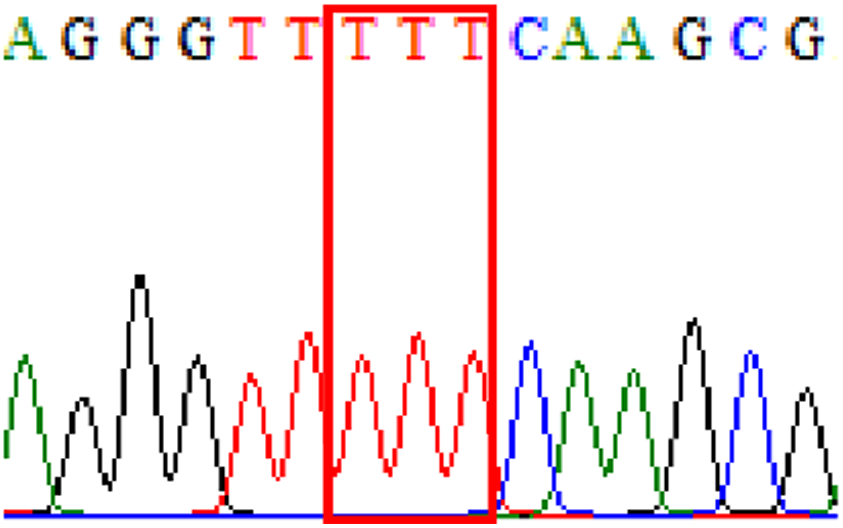	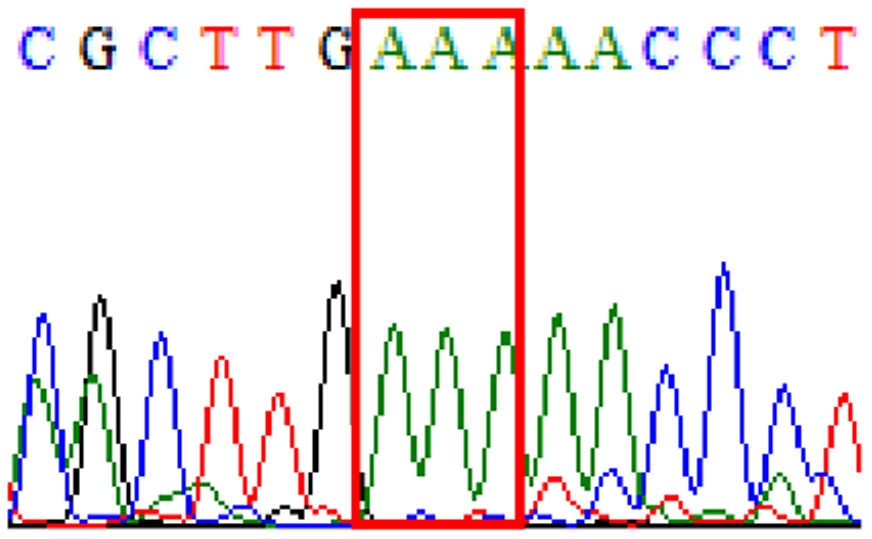	11.4% (7/61)
		D481E	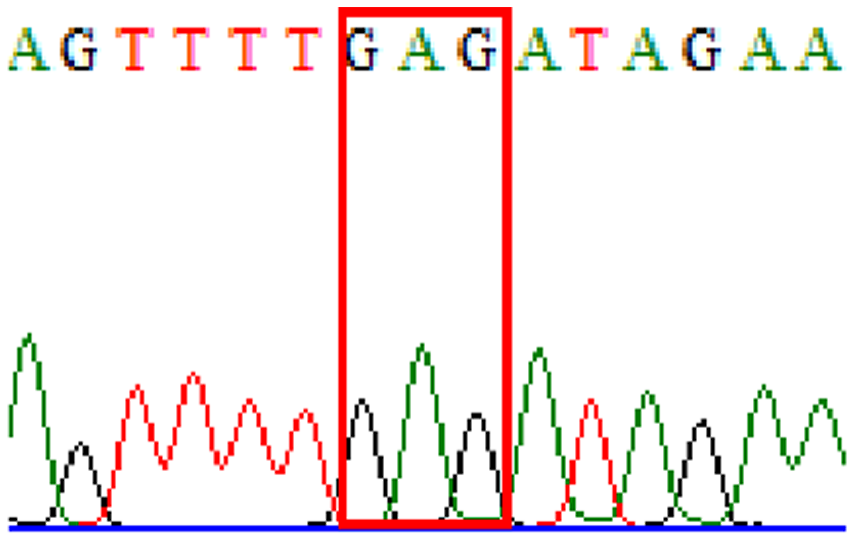	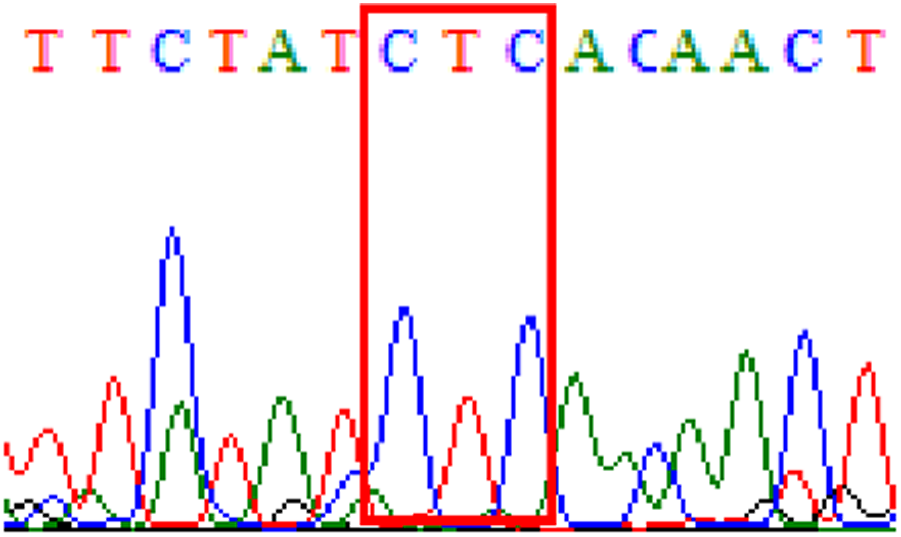	16.4% (10/61)
		R484K	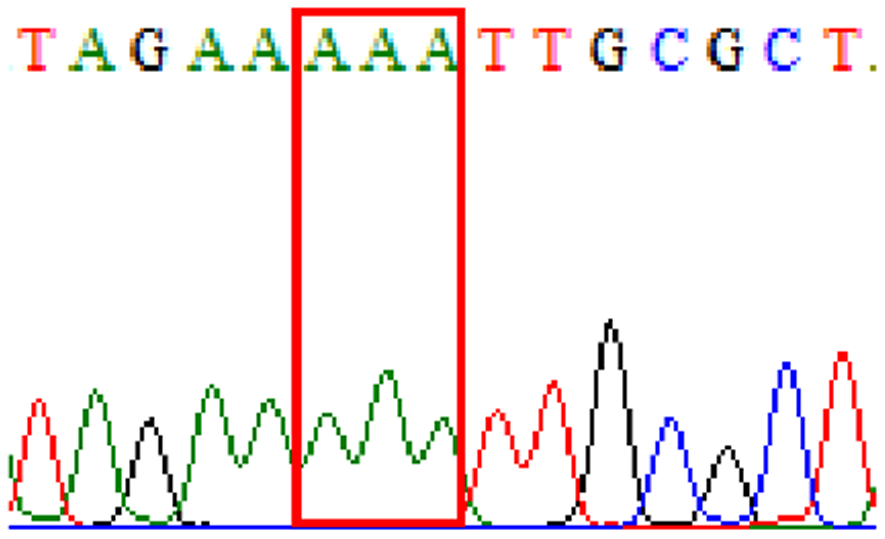	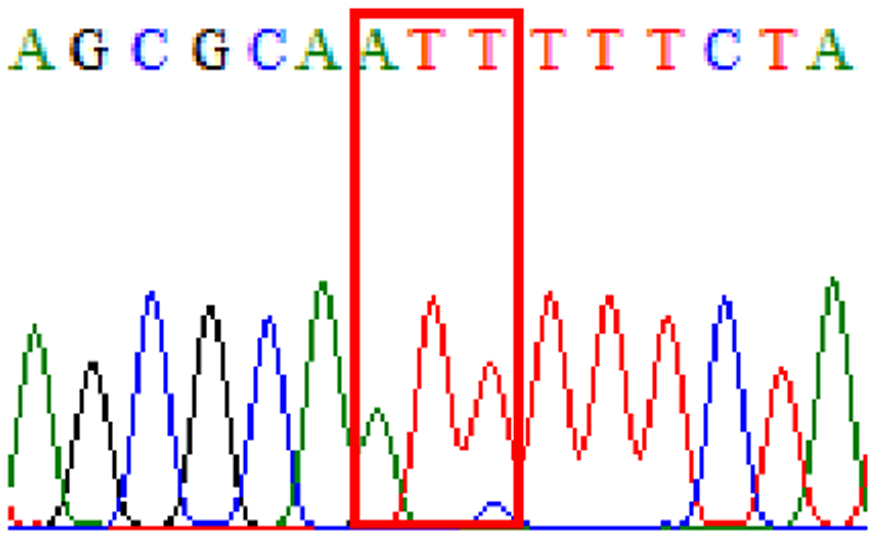	21.3% (13/61)

In this study, based on reported resistance-related mutations in 23S rRNA and *gyrA* genes, our detected resistance rates to clarithromycin was 14.8% and to levofloxacin 3.3%. Those samples demonstrated D481E and R484K in *gyrB* polymorphisms concomitantly had mutations at base triplet 87 in *gyrA* gene—46% (6/13) and 50% (5/10), respectively.

## Discussion

Determination of bacterial resistance to antibiotics is crucial to the successful treatment of any bacterial infection. This is particularly so with *H. pylori*, which affects many people in a population of a country. Monitoring of background susceptibility patterns of *H. pylori* to antibiotics allows a rational prescription of a treatment regimen for patients. This background pattern of susceptibility to antibiotics must however be continuously monitored and updated over time.

The traditional method of determining bacterial resistance has been to culture the bacterium and test it with several methods such as agar dilution, disc diffusion and E-test. However, this technique is laborious and time-consuming and requires up to a week to yield results on susceptibility to antibiotics (Mégraud & Lehours, 2007). The success of culture with *H. pylori* is also not high as the bacterium needs to be properly and quickly transported to the bacteriology laboratory (Mégraud & Lehours, 2007). In our practice, we have even resorted to direct plating of the bacteria onto special ox-blood culture media plates to improve the success of the primary culture.

In contrast, molecular methods of detecting resistance to antibiotics are a robust way of testing for resistance to an antibiotic (Mégraud et al., 2015). Collection of samples is easy from the endoscopy room where in addition to routine biopsies for the rapid urease test, biopsies are also taken and stored at 4 °C in a tube that contains a gel transport media. Bacterial DNA is well preserved in this media and remains unaltered even at ambient temperature for long periods. Results can be obtained very quickly within 3 days (from DNA extraction, DNA amplification using PCR and detection using Sanger sequencing). Molecular methods of testing for bacterial resistance are a viable and more efficient alternative to culture and antibiogram. This becomes even more so with growing requests for individual susceptibility testing for all *H. pylori* infections before treatment.

Clarithromycin and levofloxacin are two antibiotics that are commonly used in *H.pylori* treatment regimens in Malaysia as in many parts of the world. Mutations in the bacterial 23S rRNA at positions 2,142 and 2,143 are now well established for clarithromycin resistance (Mégraud et al., 2015). Although more difficult to perform, determination of mutations at key sites in topoisomerase II (DNA gyrase) or topoisomerase IV, enzymes has also been established as indicative of resistance to fluoroquinolones. As *H. pylori* lacks the topoisomerase IV enzyme, thus fluoroquinolone resistance commonly associated to point mutations in the quinolone resistance-determining region (QRDR) of the *gyrA* and *gyrB*, encoding the DNA gyrase (Miyachi et al., 2006). Thus far, *gyrB* gene has yet been well studied as the *gyrA* gene but previous reports had also described the presence of mutation in the *gyrB* gene in levofloxacin-resistant strains (Miyachi et al., 2006; Chung et al., 2012; Rimbara et al., 2012).

Previous reports showed mutations at Asn-78 and/or Asp-91 of the *gyrA* were predominantly in levofloxacin resistance (Miyachi et al., 2006; Chung et al., 2012). Intriguingly, in our study, sequencing of the *gyrA* gene revealed that 32.8% of samples carried AAC triplet at base 87 which was different from AAT triplet demonstrated by the *H. pylori* reference strains (accession numbers 26995, U27270 and L29481) available in GenBank database. This nucleotide substitution does not alter amino acid change known as silent mutation or variant at base 87. As for the *gyrB* subunit, no reported resistance-related mutation at position 463 was detected.

In our local setting in Malaysia, clarithromycin resistance has been tested for many years (Ahmad et al., 2009; Alfizah et al., 2014; Hanafiah et al., 2019; Goh et al., 1997, 2000; Ho et al., 2010; Goh & Navaratnam, 2011; Parasakthi & Goh, 1995; Teh et al., 2014). Results have consistently shown to be zero or very low (Table 3). In one study performed from our centre using molecular methods, Ho et al. (2010) showed a resistance rate of 2.9%. Later studies have shown an increase in resistance rates to 6.8% and 12.2% but they are still relatively low (Teh et al., 2014; Hanafiah et al., 2019). Clarithromycin is a key antibiotic that we continue to recommend as first-line therapy. A recently published paper from our centre has shown a high success rate with 2-week STT incorporating clarithromycin which is reflective of the susceptibility of our local strains to clarithromycin (Hwong-Ruey Leow, Chang & Goh, 2020). However, with the rise in resistance rates as shown in this present study, success rates of this therapy will drop in the future. Reports from other countries in the Asian Pacific region and Europe have already demonstrated a high as well as a rising clarithromycin resistance rate (Horiki et al., 2009; Kuo et al., 2017; Megraud et al., 2013).

**Table 3:** Primary antibiotic resistance over time in Malaysia.

Study	Year of study	Sample size (*n*)	Methodology	% Resistance to clarithromycin	% Resistance to levofloxacin
Parasakthi & Goh, 1995	1994	20	E-test	0	-
Goh et al., 1997	1996	63	E-test	0	-
Goh et al., 2000	1999	74	E-test	0	-
Ho et al., 2010	2002	107	Molecular	2.9	-
Alfizah et al., 2014	2004–2007	161	E-test	1.2	1.9
Ahmad et al., 2009	2005–2007	187	E-test	2.1	-
Goh & Navaratnam, 2011	2009	90	E-test	0	-
Teh et al., 2014	2014	102	E-test	6.8	6.8
Hanafiah et al., 2019	2014–2015	41	E-test	12.2	17.1
Present study, 2021	2019–2020	62	Molecular	14.8	3.3

**Note:**

- Not done.

Levofloxacin has also been increasingly used in clinical practice in Malaysia. We have reported its use as an effective rescue therapy previously (Goh, Manikam & Qua, 2012). Although previously reported to be low, levofloxacin resistance has also increased compared to previous reports, in the present study and this is also alarming to us. Background resistance rates to levofloxacin have been shown to have increased in several countries in the Asian Pacific region (Hu, Zhu & Lu, 2017; Liou et al., 2015; Shu et al., 2018; Lee et al., 2019a, 2019b). Nevertheless, most past studies reported that levofloxacin is mainly affected by *gyrA* but the *gyrB* mutation has a steady relationship with *gyrA* 87–91 mutations (Rimbara et al., 2012; Miyachi et al., 2006; Miftahussurur et al., 2016; Miftahussurur et al., 2019; Wang et al., 2010). Recently, Miftahussur et al. reported the detection of 48.3% (14/29) double polymorphisms (D481E and R484K) simultaneously in the *gyrB* gene in most of the garenoxacin-resistant *H. pylori* strains in Nepal and Bangladesh (Miftahussurur et al., 2019). In our study, we observed the presence of both polymorphisms D481E (16.4%) and R484K (21.3%). Hence, the relevance of these two polymorphisms for levofloxacin resistance either individually or in combination warrants further investigation in future study to confirm their clinical significance.

In this study, out of 61 samples, one sample was found to carry reported resistance-related mutations corresponding to clarithromycin (A2147G in 23S rRNA) and levofloxacin (D91N in *gyrA*) from the same patient before antibiotic treatment. However, one limitation of our study is unable to rule out whether the detection of these two mutations in a single sample containing biopsies taken from two different gastric sites is due to multiple infections or heteroresistance. Susceptible and homoresistant/heteroresistant *H. pylori* strains may co-exist at the same as well as different gastric regions. Therefore, detection of heteroresistance cases by exploring how the *H. pylori* loads differ between at least two gastric regions from the same patient or using pure isolates should be taken into account in the assessment of resistance in the future study.

Empirical therapy is widely recommended for the eradication of *H. pylori* in many countries including Malaysia with a lack of in vitro antimicrobial resistance. Unrestricted use of clarithromycin against *H. pylori* attributes to suboptimal results and antibiotic resistance. In Malaysia, a low resistance rate for clarithromycin (less than 6.8%) has been reported, therefore clarithromycin (500 mg) is continued to be used as our treatment regimens together with amoxicillin (1 g) and pantoprazole 40 mg (Qua, Manikam & Goh, 2010) or rabeprazole (20 mg) (Goh, Manikam & Qua, 2012; Leow et al., 2018). However, unsatisfactory efficacy was seen with an overall eradication rate of only 71.2% in a comparative, randomized and open-labeled study (Leow et al., 2018). In 2018, the meta-analysis of the global distribution of antibiotic resistance in *H. pylori* included 178 studies comprising 66,142 isolates from 65 countries including Malaysia showed worrisome levels of resistance rates to clarithromycin, and levofloxacin in all WHO regions (Savoldi et al., 2018). In the South-East Asia region, the pooled prevalence of clarithromycin resistance significantly increased from 13% (95% CI [4%–22%]) in 2006–2008 to 21% (95% CI [1%–42%]) in 2012–2016 (*P* < 0.001). The pooled prevalence for resistance rates for levofloxacin was 29% (95% CI [16%–42%]) in 2012–2016 (Savoldi et al., 2018).

Compared with empiric chosen treatments, molecular-based tailored therapy is a better alternative for *H pylori* eradication. In many countries, the selection of antibiotics for *H. pylori* eradication therapies is made based on the background pattern of resistance to antibiotics. It is therefore important that constant monitoring of background antibiotic resistance to *H. pylori* is carried out. Molecular methods of detecting mutation in *H. pylori* strains allows us to do this fairly easily. The low number of samples was the limitation of the present study and using a large sample size is needed for further study to provide comprehensive and up-to-date information on the antibiotic resistance of *H. pylori* in Malaysia. A further step ahead is to make available individual testing of all *H. pylori* strains particularly for treatment failures where the choice of antibiotics would be crucial.

## Supplemental Information

10.7717/peerj.11518/supp-1 Supplemental Information 1 Mutations data. Mutations on 23S rRNA ( A2147G, T2186C); mutations on *gyrA ( 87Asn, D91N) and mutations on* gyrB (438Phe, D481E, R484K)

10.7717/peerj.11518/supp-2 Supplemental Information 2 Supplementary file A2147G (23S rRNA).

10.7717/peerj.11518/supp-3 Supplemental Information 3 Supplementary file T2186C (23S rRNA).

10.7717/peerj.11518/supp-4 Supplemental Information 4 Supplementary file R484K (gyrB).

10.7717/peerj.11518/supp-5 Supplemental Information 5 Supplementary file 87Asn (gyrA).

10.7717/peerj.11518/supp-6 Supplemental Information 6 Supplementary file D91N (gyrA).

10.7717/peerj.11518/supp-7 Supplemental Information 7 Supplementary file D481E (gyrB).

10.7717/peerj.11518/supp-8 Supplemental Information 8 Supplementary file 438Phe (gyrB).

## Additional Information and Declarations

### Data Availability

The following information was supplied regarding data availability: The nucleotide sequences (*n* = 105) encompassing the mutations determined in this study are available at GenBank: 23S rRNA, mutation point A2147G: MW341345–MW341353; polymorphism point T2186C are MW341355–MW341398; *gyrA*, mutation 87Asn: MW375361, MW375363–MW375381; mutation D91N: MW375362, MW375382; *gyrB*, polymorphisms 438Phe: MW375406–MW375412; D481E: MW375383, MW375385–MW375393; and R484K: MW375384, MW375394–MW375405. The accessions are also available in the Supplemental File.
