# Supplementary material for: Current status of Helicobacter pylori resistance to Clarithromycin and Levofloxacin in Malaysia—findings from a molecular based study
Source: PeerJ. 2021 Jun 9;9:e11518. doi: 10.7717/peerj.11518 (PMC8197033; doi:10.7717/peerj.11518)
Supplement: Supplemental Information 1 — Mutations on 23S rRNA ( A2147G, T2186C); mutations on gyrA ( 87Asn, D91N) and mutations on gyrB (438Phe, D481E, R484K) [file peerj-09-11518-s001.pdf]

	No.	Sequence ID	Accession No.	Sample ID
	1	SUB8683063 Seq1	MW341345	3
	2	SUB8683063 Seq2	MW341346	28
	3	SUB8683063 Seq3	MW341347	70
	4	SUB8683063 Seq4	MW341348	109
23S rRNA A2147G	5	SUB8683063 Seq5	MW341349	124
	6	SUB8683063 Seq6	MW341350	41
	7	SUB8683063 Seq7	MW341351	60
	8	SUB8683063 Seq8	MW341352	82
	9	SUB8683063 Seq9	MW341353	118

	No.	Sequence ID	Accession No.	Sample ID
	1	SUB8683402 Seq1	MW341355	100
	2	SUB8683402 Seq2	MW341356	102
	3	SUB8683402 Seq3	MW341357	106
	4	SUB8683402 Seq4	MW341358	112
	5	SUB8683402 Seq5	MW341359	115
	6	SUB8683402 Seq6	MW341360	116
	7	SUB8683402 Seq7	MW341361	118
	8	SUB8683402 Seq8	MW341362	119
	9	SUB8683402 Seq9	MW341363	120
	10	SUB8683402 Seq10	MW341364	122
	11	SUB8683402 Seq11	MW341365	124
	12	SUB8683402 Seq12	MW341366	19
	13	SUB8683402 Seq13	MW341367	20
	14	SUB8683402 Seq14	MW341368	26
	15	SUB8683402 Seq15	MW341369	2
	16	SUB8683402 Seq16	MW341370	30
23S rRNA T2186C	17	SUB8683402 Seq17	MW341371	33
	18	SUB8683402 Seq18	MW341372	38
	19	SUB8683402 Seq19	MW341373	39
	20	SUB8683402 Seq20	MW341374	3
	21	SUB8683402 Seq21	MW341375	40
	22	SUB8683402 Seq22	MW341376	41
	23	SUB8683402 Seq23	MW341377	42
	24	SUB8683402 Seq24	MW341378	43
	25	SUB8683402 Seq25	MW341379	46
	26	SUB8683402 Seq26	MW341380	53
	27	SUB8683402 Seq27	MW341381	55
	28	SUB8683402 Seq28	MW341382	56
	29	SUB8683402 Seq29	MW341383	59
	30	SUB8683402 Seq30	MW341384	5
	31	SUB8683402 Seq31	MW341385	61
	32	SUB8683402 Seq32	MW341386	62

33	SUB8683402 Seq33	MW341387	64
34	SUB8683402 Seq34	MW341388	68
35	SUB8683402 Seq35	MW341389	70
36	SUB8683402 Seq36	MW341390	72
37	SUB8683402 Seq37	MW341391	75
38	SUB8683402 Seq38	MW341392	77
39	SUB8683402 Seq39	MW341393	7
40	SUB8683402 Seq40	MW341394	82
41	SUB8683402 Seq41	MW341395	88
42	SUB8683402 Seq42	MW341396	95
43	SUB8683402 Seq43	MW341397	96
44	SUB8683402 Seq44	MW341398	9

	No.	Sequence ID	Accession No.	Sample ID
gyrA 87Asn	1	BankIt2406531 Seq1	MW375361	130
	2	BankIt2406964 Seq1	MW375363	100
	3	BankIt2406964 Seq2	MW375364	102
	4	BankIt2406964 Seq3	MW375365	106
	5	BankIt2406964 Seq4	MW375366	115
	6	BankIt2406964 Seq5	MW375367	124
	7	BankIt2406964 Seq6	MW375368	21
	8	BankIt2406964 Seq7	MW375369	22
	9	BankIt2406964 Seq8	MW375370	30
	10	BankIt2406964 Seq9	MW375371	32
	11	BankIt2406964 Seq10	MW375372	39
	12	BankIt2406964 Seq11	MW375373	40
	13	BankIt2406964 Seq12	MW375374	42
	14	BankIt2406964 Seq13	MW375375	46
	15	BankIt2406964 Seq14	MW375376	59
	16	BankIt2406964 Seq15	MW375377	5
	17	BankIt2406964 Seq16	MW375378	61
	18	BankIt2406964 Seq17	MW375379	64
	19	BankIt2406964 Seq18	MW375380	66
	20	BankIt2406964 Seq19	MW375381	69

	No.	Sequence ID	Accession No.	Sample ID
gyrA D91N	1	BankIt2406532 Seq1	MW375362	2
	2	BankIt2406971 Seq1	MW375382	60

gyrB 438Phe

No.	Sequence ID	Accession No.	Sample ID
1	BankIt2406536 Seq1	MW375406	21
2	BankIt2406986 Seq1	MW375407	103
3	BankIt2406986 Seq2	MW375408	105
4	BankIt2406986 Seq3	MW375409	109
5	BankIt2406986 Seq4	MW375410	32
6	BankIt2406986 Seq5	MW375411	61
7	BankIt2406986 Seq6	MW375412	80

gyrB D481E

No.	Sequence ID	Accession No.	Sample ID
1	BankIt2406537 Seq1	MW375383	5
2	BankIt2406999 Seq1	MW375385	102
3	BankIt2406999 Seq2	MW375386	19
4	BankIt2406999 Seq3	MW375387	22
5	BankIt2406999 Seq4	MW375388	26
6	BankIt2406999 Seq5	MW375389	30
7	BankIt2406999 Seq6	MW375390	55
8	BankIt2406999 Seq7	MW375391	61
9	BankIt2406999 Seq8	MW375392	75
10	BankIt2406999 Seq9	MW375393	95

gyrB R484K

No.	Sequence ID	Accession No.	Sample ID
1	BankIt2406539 Seq1	MW375384	5
2	BankIt2407008 Seq1	MW375394	102
3	BankIt2407008 Seq2	MW375395	116
4	BankIt2407008 Seq3	MW375396	19
5	BankIt2407008 Seq4	MW375397	22
6	BankIt2407008 Seq5	MW375398	26
7	BankIt2407008 Seq6	MW375399	30
8	BankIt2407008 Seq7	MW375400	55
9	BankIt2407008 Seq8	MW375401	61
10	BankIt2407008 Seq9	MW375402	68
11	BankIt2407008 Seq10	MW375403	69
12	BankIt2407008 Seq11	MW375404	75
13	BankIt2407008 Seq12	MW375405	95
